# Relationship Between Anemia and Systemic Inflammation in People Living With HIV and Tuberculosis: A Sub-Analysis of the CADIRIS Clinical Trial

**DOI:** 10.3389/fimmu.2022.916216

**Published:** 2022-06-23

**Authors:** Mariana Araújo-Pereira, Beatriz Barreto-Duarte, María B. Arriaga, Laura W. Musselwhite, Caian L. Vinhaes, Pablo F. Belaunzaran-Zamudio, Adam Rupert, Luis J. Montaner, Michael M. Lederman, Irini Sereti, Juan G. Sierra Madero, Bruno B. Andrade

**Affiliations:** ^1^Instituto Gonçalo Moniz, Fundação Oswaldo Cruz, Salvador, Brazil; ^2^Multinational Organization Network Sponsoring Translational and Epidemiological Research (MONSTER) Initiative, Salvador, Brazil; ^3^Programa de Pós-Graduação em Patologia Humana e Experimental, Universidade Federal da Bahia, Salvador, Brazil; ^4^Programa de Pós-Graduação em Clínica Médica, Universidade Federal do Rio de Janeiro, Rio de Janeiro, Brazil; ^5^Curso de Medicina, Universidade Salvador (UNIFACS), Salvador, Brazil; ^6^Instituto de Medicina Tropical Alexander Von Humboldt, Universidad Peruana Cayetano Heredia, Lima, Peru; ^7^Department of Solid Tumor Oncology, Levine Cancer Institute, Charlotte, NC, United States; ^8^Bahiana School of Medicine and Public Health, Bahia Foundation for the Development of Sciences, Salvador, Brazil; ^9^Infectious Diseases Department, Instituto Nacional de Ciencias Médicas y Nutrición Salvador Zubirán, Mexico City, Mexico; ^10^National Institutes of Allergy and Infectious Diseases, National Institutes of Health, Bethesda, MD, United States; ^11^The Wistar Institute, Philadelphia, PA, United States; ^12^Division of Infectious Diseases and HIV Medicine, Department of Medicine, Case Western Reserve University, Cleveland, OH, United States

**Keywords:** HIV, Tuberculosis, inflammation, degree of inflammatory perturbation, anemia

## Abstract

People with HIV (PWH) are at increased risk of developing active tuberculosis (TB), and anemia is a common complication in both conditions. Anemia in TB patients has been linked to immune activation, levels of inflammatory biomarkers in blood, and risk for HIV disease progression and death. In this study we show that anemia was associated with a more pronounced inflammatory profile in HIV-TB coinfected persons in a cohort of 159 individuals with advanced HIV disease (CD4 count < 100 cells/µL) recruited as part of a randomized clinical trial (NCT00988780). A panel of plasma biomarkers was assessed on plasma obtained prior to combination antiretroviral therapy (cART) initiation. We performed a series of multidimensional analyses including clinical variables and concentrations of inflammatory biomarkers to profile systemic inflammation of PWH with and without anemia. We observed that TB participants presented with moderately lower levels of hemoglobin than non-TB participants. These participants also presented a higher Degree of Inflammatory Perturbation (DIP) score, related to increased levels of IFN-γ and TNF. The DIP was associated with TB coinfection and anemia before cART initiation. Future mechanistic studies are warranted to assess the determinants of such associations and the implications on treatment outcomes.

## Introduction

By the end of 2020 it was estimated that there were 38 million people with HIV (PWH) globally. Every year approximately 1.7 million people are newly infected and about 690,000 people die from complications caused by HIV infection (1). When not treated, PWH typically progress through stages, the last being the acquired immunodeficiency syndrome (AIDS) (2).

AIDS is defined by a CD4+ T-cell count below 200 cells/µL or by the identification of at least one AIDS-defining illness. Tuberculosis (TB) is among the most frequently diagnosed AIDS-defining illnesses (2). TB is an infectious disease caused by Mycobacterium tuberculosis (*Mtb*), and in 2019, approximately 10 million people were living with active TB (PLTB) and 1.3 million TB-associated deaths were estimated. Also according to these statistics, 8% of PLTB are thought to be co-infected with HIV, and 16% of TB-associated deaths occurred in this population of HIV-TB co-infected persons (1).

PWH coinfected with TB have a substantially higher death risk (3), a lower quality of life and health (4), and lower hemoglobin (Hb) concentrations in peripheral blood (5), compared to mono-infected patients (i.e. either TB or HIV alone). Low levels of Hb are associated with increased levels of inflammatory biomarkers in blood of TB patients (6) and with the acceleration of HIV disease progression (7, 8). A previous study of our group had demonstrated, using routine clinical laboratory parameters, that anemia is a risk factor for unfavorable anti-TB treatment outcomes, and that Hb levels are associated with a heightened degree of inflammatory perturbation in TB-HIV patients (9). In the present study, we expand the investigation of inflammatory disturbance associated with anemia in the context of TB-HIV by examining levels of inflammatory cytokines using plasma samples of PWH with advanced disease participating in a randomized clinical trial (10). Our aim was to evaluate the relationship of low Hb levels and the inflammatory profile of PWH and AIDS-TB.

## Methods

### Study Outline

The CADIRIS study (CCR5 Antagonism to Decrease the Incidence of immune reconstitution inflammatory syndrome (IRIS) in HIV infected participants, NCT00988780) enrolled 276 participants from 2009 to 2012 at five clinical sites in Mexico and South Africa in a double-blind, randomized, placebo-controlled study that followed participants for 1 year after cART initiation (10). Participants were adults (age at least 18 years) with HIV-infection, cART-naïve, and with CD4 cell count equal to or lower than 100 cells/µL; individuals with Hb <8g/dL were excluded. For this sub-study, we included CADIRIS participants with AIDS-defining illnesses or HIV-associated wasting syndrome at enrollment, and with available blood samples for analysis (n=159). We evaluated data of nationality, sex, TB status (active TB or no TB), anemia severity (healthy, mild and moderate), and levels of biomarkers measured in cryopreserved plasma specimens from study participants collected at baseline (pre-cART).

### Ethical Statement

CADIRIS study was approved by the Ethics Review Committee at all participating institutions. All participants provided written informed consent before any study procedures.

### Definitions

Anemia was defined according to World Health Organization (WHO) guideline criteria as levels of Hb below (<)13 g/dL for men or <12 g/dL for women (11). Mild anemia was defined as Hb >10 g/dL and <13 g/dL for men; and >10 and <12 g/dL for women, whereas moderate anemia was defined as Hb>8 g/dL and <=10 g/dL for both sexes. Severe anemia (Hb<8g/dL) was an exclusionary criterion as previously stated. TB diagnoses were made with mycobacterial blood culture, tuberculin skin tests, and chest radiographs.

### Biomarker Measurement

Collection and cryopreservation of blood derived specimens have been described previously (12). We measured biomarkers using commercial kits to evaluate C-Reactive Protein (CRP), Interferon-γ (IFN-γ), interleukin (IL)-1β, IL-6, IL-8, IL-10, IL-12p70, IL-17, interferon-inducible protein (CXCL) 10, P-selectin, serum amyloid A (SAA), tumor necrosis factor-α (TNF), Leukotriene B4 (LTB4), soluble (s)CD14, sCD40 ligand, sCD163, Von Willebrand Factor (vWF), fibrinogen (FIB), proteins C and S, Hyaluronic Acid (HA), D dimer and 25-hydroxyvitamin-D levels, using electrochemiluminescence (MESO scale discovery), enzyme-linked immunosorbent assays (R&D Systems, AdipoBioscience, Zymutest, ALPCO), enzyme-linked fluorescent assay (Biomeriuex) and enzyme coagulation kit (Corgenix) methods.

### Statistical Analysis

Descriptive statistics were used to present data, using median values with interquartile ranges (IQR) as measures of centrality and dispersion for continuous variables. Categorical variables were described using frequency (no.) and proportions (%). The Pearson’s chi-square test was used to compare categorical variables between study groups (i.e. TB and non-TB). The Mann–Whitney U test (for two unmatched groups; i.e. TB and non-TB) was used to compare continuous variables. The Spearman rank test was used to assess correlations between Hb and biomarkers.

The degree of inflammatory perturbation (DIP) was calculated to identify the general inflammatory environment of the participants. DIP was based on the molecular degree of perturbation (MDP) (13) and calculated as previously described (9). Details about this method are described in **Supplementary Figure 1**.

We used backward stepwise linear regression to examine the association between biomarkers and Hb levels. All biomarkers were included in this analysis and all values were log 2 transformed. Next, we used multi-level Poisson regression models to estimate the association between units of biomarkers and Hb with DIP score levels. The results were presented in the form of adjusted Odds Ratio (aOR) and 95% confidence intervals (CI).

Differences with p-values below 0.05 after adjustment for multiple comparisons (Holm-Bonferroni) were considered statistically significant. The statistical analyses were performed using IBM SPSS version 25; and R (version 4.4.1), using mdp (version 1.8.0), rstatix (version 0.4.0), stats (version 3.6.2), metaphor (version 2.4.0) and questionr (version 0.7.1) R packages.

## Results

### Characteristics of the Study Participants

We included 159 PWH from Mexico and South Africa, mostly men (74.8%) in both countries. All participants were diagnosed with an AIDS-defining illness detailed in **Supplementary Table 1**. We stratified these participants into two main groups: non-TB (n=98) and TB (n=61). We found that 65.6% of participants with TB were from South Africa, while 78.6% of the non-TB group were from Mexico (p<0.001). TB participants were more frequently self-reported Blacks, whereas “Mixed” was the race most often self-declared in non-TB participants (p<0.001) (**Table 1**).

**Table 1:** Characteristics of the study population.

	ALL (n=159)	non-TB (n=98)	TB (n=61)	P-value
**Country, no. (%):**				**<0.001**
**Mexico**	98 (61.6)	77 (78.6)	21 (34.4)	
**South Africa**	61 (38.4)	21 (21.4)	40 (65.6)	
**Male, no. (%):**	119 (74.8)	76 (77.6)	43 (70.5)	0.418
**Race, no. (%):**				**<0.001**
**Asian**	1 (0.63)	1 (1.02)	0 (0.00)	
**Black**	58 (36.5)	18 (18.4)	40 (65.6)	
**Mixed**	88 (55.3)	70 (71.4)	18 (29.5)	
**White**	12 (7.55)	9 (9.18)	3 (4.92)	
**Hemoglobin (g/dL), median (IQR):**	12.0 (10.8-13.2)	12.2 (11.3-13.6)	11.3 (9.80-13.0)	**0.002**

**HIV Viral Load log10, median (IQR):**	5.27 (4.93-5.62)	5.40 (5.00-5.76)	5.51 (5.15-5.82)	0.181
**CD4 (cells/μL), median (IQR):**	31 (16–58)	31 (14–53)	32 (21–62)	0.210
**CD8 (cells/μL), median (IQR):**	475 (342–760)	524 (385–800)	423 (296–671)	**0.037**
**CD4/CD8, median (IQR):**	0.06 (0.03-0.10)	0.05 (0.02-0.10)	0.07 (0.05-0.13)	**0.004**

Bold font indicates statistical significance at p<0.05. Data are shown as number and frequency (percentage). Data were compared between groups using the Pearson chi-square test for categorical variables and the Wilcoxon test for continuous variables.

IQR, interquartile range.

TB participants presented with lower levels of Hb with a median of 11.3 g/dL (9.80-13.0) in comparison to non-TB participants, with 12.2 g/dL (11.3-13.6) (p=0.002). Of note, this difference was observed comparing all participants according to TB status, as well as comparing TB status according to sex (non-TB male: 12.2(11.1-13.8) *vs* TB male: 11.8(10.3-13.2), p = 0.081; non-TB female: 12.4(11.5-12.7) *vs* TB female: 10.8 (9.57-11.2), p=0.005). Interestingly, TB participants had lower CD8 T cell counts (p = 0.037) and higher CD4/CD8 ratio values when compared to the non-TB group (p=0.004). Baseline plasma HIV levels and CD4 T-cell counts were similar in the groups (**Table 1**).

**Figure 1:**
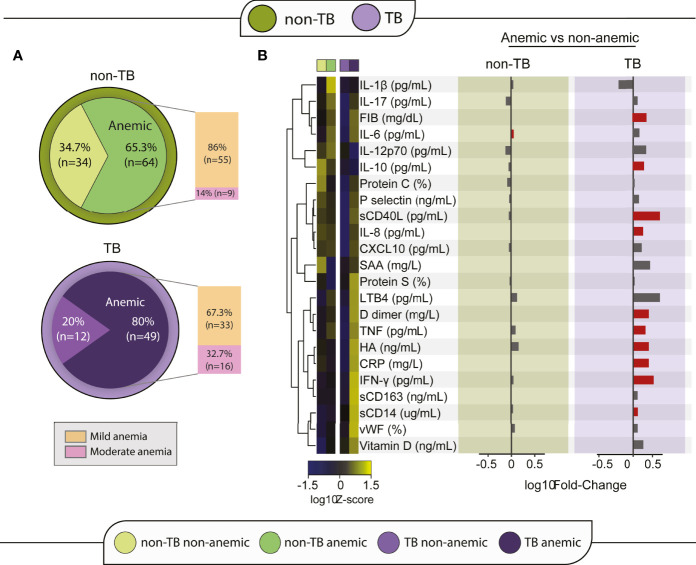
Anemia is linked to a differential inflammatory profile in TB-HIV participants. **(A)** Participants of each group were divided according to anemia status and grade. To define anemia grade, the cut-off point for hemoglobin of 12 g/dL for women and 13 g/dL for men was used to define normal levels, the cut-off of 10 g/dL and lower than normal levels was used for mild anemia and hemoglobin between 8 and 10 g/dL was classified as moderate anemia. **(B)** A heatmap was designed to depict the overall pattern of inflammatory markers. A one-way hierarchical cluster analysis (Ward’s method) was performed. Dendrograms represent Euclidean distance. Right panel: A log10 of fold-change was calculated and statistical analyses were performed using the Mann–Whitney U adjusted test. Significant differences (p < 0.05) between groups are highlighted in red bars.

We decided to examine how participants were distributed in terms of anemia status and we characterized their inflammatory profile. About 65% (n=64) of the non-TB group presented with anemia, whereas in the TB group this frequency was higher, at 80% (n=49). Noteworthy, we found no difference in occurrence of anemia when comparing patients from Mexico and South Africa. Most anemic participants in both groups, had mild anemia (non-TB: 86%; TB: 67.3%). Participants in the TB group exhibited a higher proportion of moderate anemia (33%) than participants in the non-TB group (14%, p=0.007) (**Figure 1A**).

### Anemia Is Linked to the Inflammatory Profile in TB

After classifying the participants according to anemia status, the biomarker levels of the anemic and non-anemic participants were compared within each clinical group, to identify the relationship of anemia to systemic inflammation. A hierarchical heatmap was built using log2-transformed and Z-score normalized data and clustered according to anemia status to analyze the differences of inflammatory profile between groups and conditions (**Figure 1B**). In the non-TB group, anemia appeared not to be significantly linked to levels of inflammatory cytokines, except for IL-6 levels which were increased in anemic participants, with a median of 2.45 pg/mL (IQR:1.68-3.64) in comparison to levels in non-anemic participants (2.14 [IQR:1.29-2.63], p = 0.046) (**Figure 1B**; **Supplementary Table 2**). In contrast, anemic participants in the TB group presented an inflammatory profile distinct from that of non-anemic participants, with significant increases in FIB, IL-10, sCD40L, IL-8, D Dimer, TNF, HA, CRP, IFN-γ and sCD14 (**Figure 1B**) levels. Medians and p-values of these comparisons are detailed in **Supplementary Table 2**.

**Figure 2:**
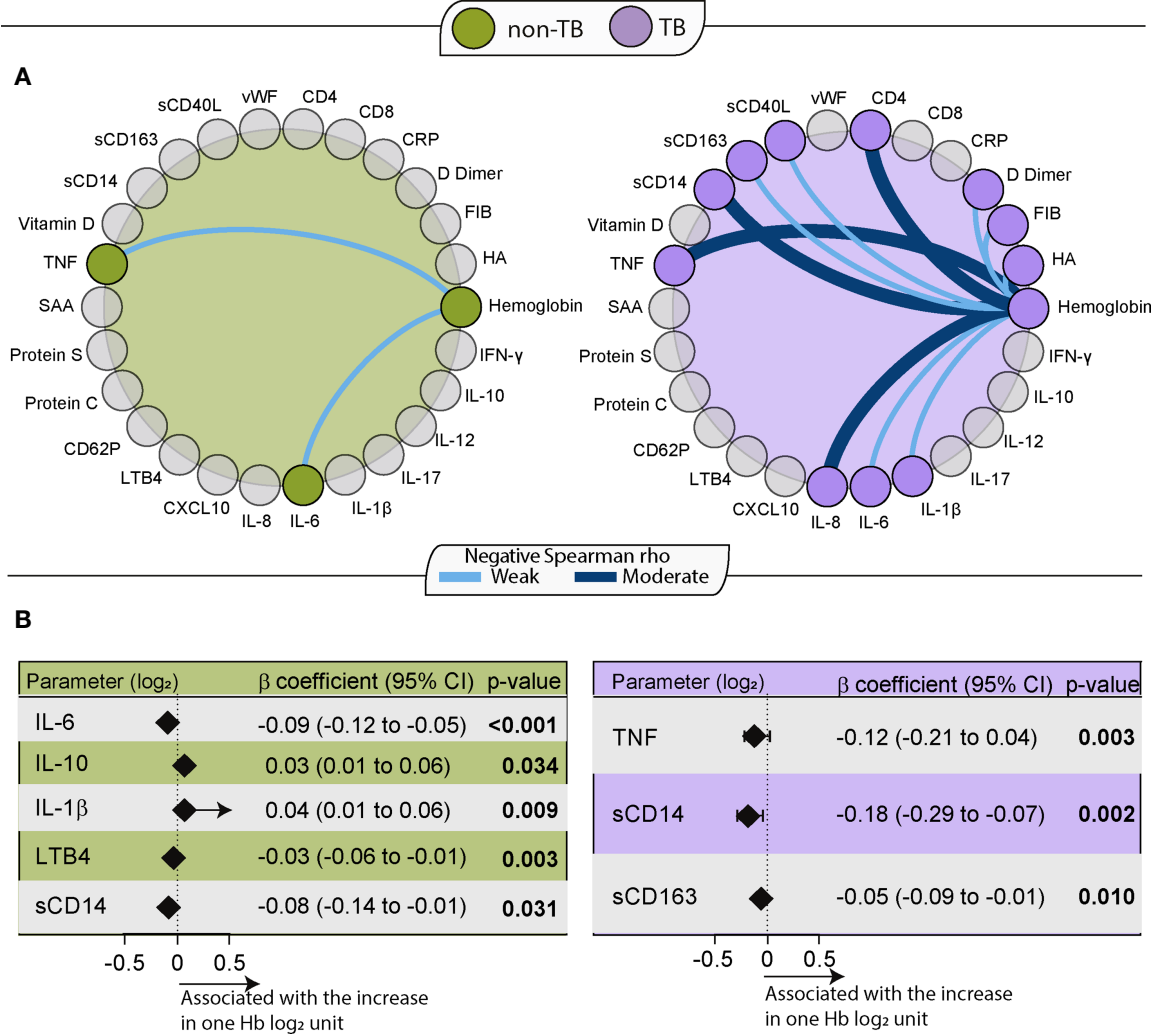
Hemoglobin levels are related to the inflammatory profile of HIV-TB participants. **(A)** Spearman correlation test between Hb levels and laboratory measurements for each group (Green: non-TB; Purple: TB). Light blue lines indicate a weakly negative (rho <0.40) correlation, and dark blue lines indicate a moderately strong negative (0.4≤ rho <0.6) correlation between the linked parameters. All correlations in this chart had p value less than 0.05. **(B)** Backward stepwise linear regression to identify biomarker measurements independently associated with Hb levels. All parameters were log2 transformed. Only significant parameters in the last step are shown. Left: non-TB (green). Right: TB (purple).

Next, a Spearman correlation network analysis between Hb concentrations and inflammatory marker levels was performed for each group (non-TB and TB). CD4 and CD8 T-cell counts were also included in the analysis. Hemoglobin values displayed negative correlations with inflammatory markers in both groups, being more frequently identified in the TB group (**Figure 2**).

In the non-TB group, significant albeit weak correlations were noted between Hb and two markers: TNF (r=-0.21, p=0.03) and IL-6 (r=-0.22, p=0.03). In the TB group correlations were found between Hb and IL-1β (r=-0.28, p=0.04), IL-6 (r=-0.29, p=0.04), sCD163 (r=-0.3, p=0.04), sCD40L (r=-0.32, p=0.02), FIB (r=-0.36, p=0.007), HA (r=-0.38, p=0.005), D Dimer (r=-0.39, p=0.005), IL-8 (r=-0.44, p=0.001), sCD14 (r=-0.47, p<0.001) and TNF (r=-0.51, p<0.001). Of note, in the TB group the negative correlation of Hb was also seen with the CD4 count (r=-0.5, p<0.001). Moreover, the correlation between Hb values and CD8 count was not statistically significant (r=0.068; p=0.06) (**Figure 2A**).

A network analysis between biomarkers in groups stratified according to TB and anemia was also performed. In this analysis, it was observed that CRP was the most interconnected marker with other markers among all groups, but especially in anemic TB participants. In this group, there was also a high amount of cytokine connectivity with IL-6. This greater interconnectivity in HIV-TB participants with anemia reflects a greater network density compared to the other groups and may be associated with a coordinated immune response (**Supplementary Figure 2**).

When we performed a backward stepwise linear regression to identify the independent associations between cytokine measurements and Hb values in the two main study groups, we found that higher values of pro-inflammatory cytokines were associated with low Hb values in both groups (non-TB and TB) (**Figure 2B**). In the non-TB group, decreasing levels of IL-6 (β coefficient: -0.09; 95%CI: -0.12 to -0.05; p<0.001), LTB4 (β coefficient: -0.03; 95%CI: -0.06 to -0.01; p=0.003) and sCD14 (β coefficient: -0.08; 95%CI: -0.14 to -0.01; p=0.031) as well as increasing levels of IL-10 (β coefficient: 0.03; 95%CI: 0.01 to 0.06; p=0.034) and IL-1β (β coefficient: 0.04; 95%CI: 0.01 to 0.06; p=0.009) were independently associated with an increase of one Hb (log2) unit. In the TB group, decreasing levels of TNF (β coefficient: -0.12; 95%CI: -0.21 to 0.04, p=0.003), sCD14 (β coefficient: -0.18; 95%CI: -0.29 to -0.07, p=0.002) and sCD163 (β coefficient: -0.05 95%CI: -0.09 to -0.01, p=0.01) were associated with an increase of one Hb (log2) unit. These associations indicate that, in PWH, low Hb levels are associated with greater systemic inflammation.

### PWH With Anemia Have Higher Degrees of Inflammatory Perturbation Even in the Absence of TB

**Figure 3:**
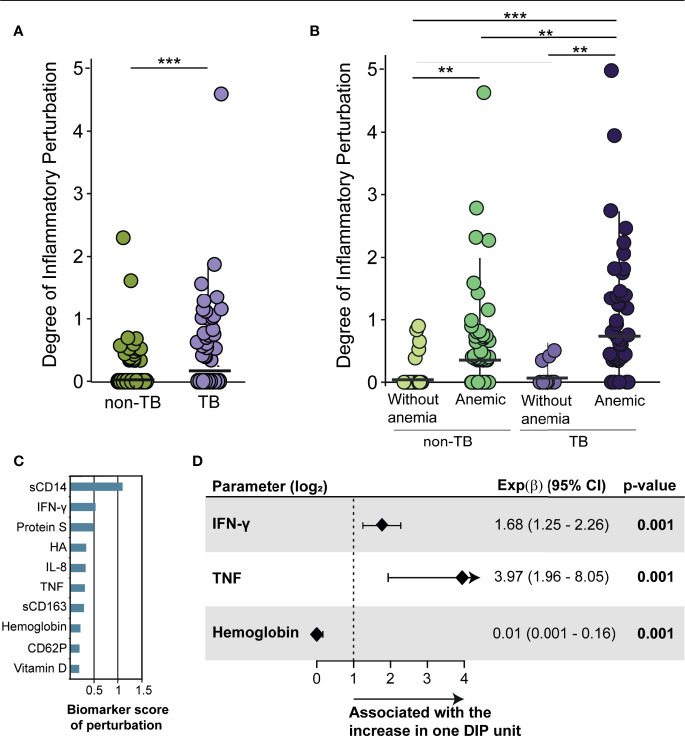
The degree of inflammatory perturbation increases in anemic participants. **(A)** Scatter plots of the degree of inflammatory perturbation (DIP) by sample in the main groups: non-TB (green) and TB (purple); **(B)** Scatter plots of DIP by sample in each group: non-TB non-anemic, used as control (light green); non-TB anemic (dark green); TB non-anemic (light purple); and TB anemic (dark purple). DIP score values were compared between groups according to TB and anemia status. Lines in the scatter plots represent median values and data were compared using the Mann–Whitney U test. **p < 0.01; ***p < 0.001. **(C)** Top 10 biomarker score of perturbation in the comparison non-TB vs TB. The score was obtained using DIP approach. **(D)** Poisson regression to identify biomarker measurements independently associated with increases of DIP score in HIV-TB participants. All parameters are log2 transformed. Only significant parameters are shown.

With the individuals classified according to TB infection status in two main groups, as well as subdivided accordingly to their anemia status in four groups (non-TB non-anemic, non-TB anemic, TB non-anemic and TB anemic), we employed DIP approach, to estimate the overall degree of inflammation and/or immune activation in TB participants. In the first comparison, non-TB participants were used as controls (**Figure 3A**). Thus, it was possible to observe that TB coinfection is linked to an increased DIP (p<0.001) in PWH (**Figure 3A**).

We next assessed whether the groups with anemia had a different DIP than those without anemia, using the non-TB non-anemic group as control (**Figure 3B**). First, it was observed that TB non-anemic participants had the same low profile of perturbation as did the control group (p=0.810). DIP values were elevated in anemic participants without (p=0.004) and with TB (p=0.004) when compared to their respective non-anemic peers. The anemic TB group had a higher median DIP value than the non-anemic TB group (p=0.001), arguing that anemia in HIV-TB context is associated with inflammatory disturbance/activation (**Figure 3B**).

The top 10 biomarkers that contributed to inflammatory perturbation were: sCD14, IFN-γ, Protein S, HA < IL-8, TFN, sCD163, Hb, CD62P and vitamin D (**Figure 3C**). By performing Poisson regression in order to identify factors associated with higher DIP, we found that an increase in one unit of each DIP score was associated with 1 log increases in plasma IFN-γ (Exp(β): 1.68; 95%CI: 1.25-2.26, p=0.001) and TNF (Exp(β): 3.97; 95%CI: 1.96-8.06, p=0.001), as well as decreases in Hb levels (Exp(β): 0.01; 95%CI:0.001-0.16, p=0.001) in TB participants (**Figure 3D**), enabling us to identify the main markers involved in inflammatory disturbance in TB participants and, also associating low hemoglobin levels with this perturbation.

## Discussion

In this retrospective analysis of a multicenter prospective study, we evaluated the relationship between low levels of hemoglobin on the inflammatory profile in PWH and TB. We performed a comprehensive investigation, measuring numerous host soluble inflammatory mediators collected prior to antiretroviral therapy initiation, in persons with advanced HIV infection.

We found that participants with TB coinfection presented with lower Hb levels and were more often anemic than non-TB participants. This finding corroborates those of an Ethiopian cohort of 230 participants, where HIV-TB participants also had lower levels of Hb than did non-TB participants (14). In our cohort, the frequency of anemia in TB participants was 80%. This frequency was similar to the findings of an earlier study by our group, where in a Brazilian cohort with HIV-TB, 84% of participants were anemic at baseline (9).

Stratifying each main group according to anemia status, we observed that in TB, anemic participants had a distinct inflammatory profile in comparison to non-anemic participants. Other investigations have reported a higher inflammatory profile in TB participants in comparison to non-TB participants, showing that the increased systemic inflammation can be associated with a higher risk of unfavorable outcomes in HIV-TB (9, 14).

In TB participants, Hb measurements were strongly and negatively related to CD4 T-cell counts. The negative correlation with CD4 counts, interestingly, is contrary to what is reported in the literature, where a positive correlation between Hb and CD4 levels is described (15, 16). We hypothesize that in people with AIDS (or PWH and advanced disease), there may be opportunistic infections that affect the white blood cell count, which in turn can affect the CD4 T-cell count value. It has already been described that chronic diseases, infections and drug use can cause this effect among PWH (17).

Measurements of most of these inflammatory markers, especially TNF, sCD14, IL-8 and IL-6 also were strongly and negatively correlated with Hb levels. All these factors have already been described associated with a pro-inflammatory condition in PWH and reported as risk factors for the progression of HIV infection and increased morbidity and mortality (18–21). Of note, these markers identified in our data, in addition to Hb, were used to develop a composite score to predict mycobacterial IRIS and death in PWH in a separate observational cohort study (22).

In the linear regression performed here, higher levels of TNF and sCD14 appeared again associated with low Hb levels, with the addition of sCD163, which also presents this pattern in HIV-TB. TNF also was associated with increases in DIP in HIV-TB participants. TNF is potent inflammatory molecules primarily secreted by macrophages and required for control of growth of *Mtb* (23), however, this same cytokine is known to activate HIV replication in macrophages. Thus, TNF inhibits *Mtb* growth while enhancing HIV replication (24). Linking this information to our results in anemic participants, we can infer that in PWH with anemia, there is an exacerbation of TNF production. Whether anemia itself is directly causing a boost in TNF production or if augmented inflammation is leading to the establishment and/or persistence of anemia cannot be determined with our data and warrants further mechanistic investigations.

The increased concentrations of sCD14 and sCD163 likely indicate an activation of monocyte/macrophages. sCD14 is a marker of monocyte response, described as an independent predictor of mortality (21) in PWH, whereas CD163 is an important surface marker expressed on monocyte-macrophage lineage cells and shed in soluble form during inflammation (25). In PWH, Hb levels have been associated with monocyte activation, reflecting in an increased risk of inflammatory events such as atherosclerosis (26).

There are limitations to our study. One of the inclusion criteria for CADIRIS was Hb >8g/dL, effectively excluding severe anemia which might have provided better power to related Hb with other markers, as well as having an impact on regression analyses. Baseline biomarker measurement allowed for the investigation of inflammatory profile in the context of TB and anemia but did not allow us to evaluate temporal changes during treatment. Moreover, we did not evaluate latent TB infection or co-infection with helminths in these patients, even is know that this can impact in immune response. It is also important to note that TB was more frequent in participants in South Africa than among those in Mexico. Of note, in a previous study, our group demonstrated that the different inflammatory profiles of the participants were associated with their country of origin (12). This could be explained by a higher incidence of HIV-TB among participants as reported by WHO (577 vs. <1 per 100 000 cases in 2009), which is consistent with distribution of TB-cases in this study (27). Although the TB prevalence observed here was higher in South Africa compared to that in Mexico, we found no difference in occurrence of anemia between the study participants stratified by country. Africa is known for being an epicenter of a number of hemoglobinopathies while Mexico is not, and thus one could infer that such difference could underlie discrepancies in anemia and its related information. Our study did not explore the impact of conditions affecting Hb metabolism and consequently further studies are warranted to test this hypothesis.

The present study demonstrates that there is an association between lower Hb concentration and augmented inflammatory disturbance in PWH and advanced disease regardless of TB. The inflammatory activation, characterized in our paper by increased levels of TNF and IFN-γ, and low Hb levels are described in the literature as a risk factor for adverse treatment outcomes of HIV and TB separately, and our study demonstrates that both factors are often present in TB coinfected participants. Such associations described here between soluble markers in peripheral blood and anemia may underlie the pathogenesis of advanced HIV which may drive unfavorable disease outcomes.

## Data Availability Statement

The raw data supporting the conclusions of this article will be made available by the authors, without undue reservation.

## Ethics Statement

CADIRIS study was approved by the Ethics Review Committee at all participating institutions. The patients/participants provided their written informed consent to participate in this study.

## Author Contributions

MA-P, PB-Z, ML, ISe, and BA designed the study. LM, AR, ISe, and JS-M collected the data. MA-P, BB-D, MA and BA analyzed and interpreted the data. MA-P, BB-D and BA drafted the manuscript. PB-Z, LJM, ML, ISe, and BA critically revised the manuscript. ML and JS-M obtained the funding. JS-M provided administrative, technical, and material support and supervised the study. All authors contributed to manuscript revision, read, and approved the submitted version.

## Funding

The trial was funded as investigator-initiated research by Pfizer Inc, New York, NY, USA. The funder was not involved in the study design, collection, analysis, interpretation of data, the writing of this article or the decision to submit it for publication. The work of I. Sereti was supported by the Intramural Research Program of NIAID/NIH. BA is a senior scientist from the Conselho Nacional de Desenvolvimento Científico e Tecnológico (CNPq), Brazil. MA-P and BB-D received a fellowship from Coordenação de Aperfeiçoamento de Pessoal de Nível Superior (Finance code: 001). MA received a research fellowship from the Fundação de Amparo à Pesquisa do Estado da Bahia (FAPESB), Brazil.

## Conflict of Interest

JS-M reports grants from BMS, Pfizer, MSD, and Stendhal outside the submitted work. LJM reports grants from Merck and Gene-One.

The remaining authors declare that the research was conducted in the absence of any commercial or financial relationships that could be construed as a potential conflict of interest.

## Publisher’s Note

All claims expressed in this article are solely those of the authors and do not necessarily represent those of their affiliated organizations, or those of the publisher, the editors and the reviewers. Any product that may be evaluated in this article, or claim that may be made by its manufacturer, is not guaranteed or endorsed by the publisher.
