# Supplementary material for: Relationship Between Anemia and Systemic Inflammation in People Living With HIV and Tuberculosis: A Sub-Analysis of the CADIRIS Clinical Trial
Source: Front Immunol. 2022 Jun 23;13:916216. doi: 10.3389/fimmu.2022.916216 (PMC9260499; doi:10.3389/fimmu.2022.916216)
Supplement: Supplementary file 1 [file DataSheet_1.docx]

***Supplementary Material***

**
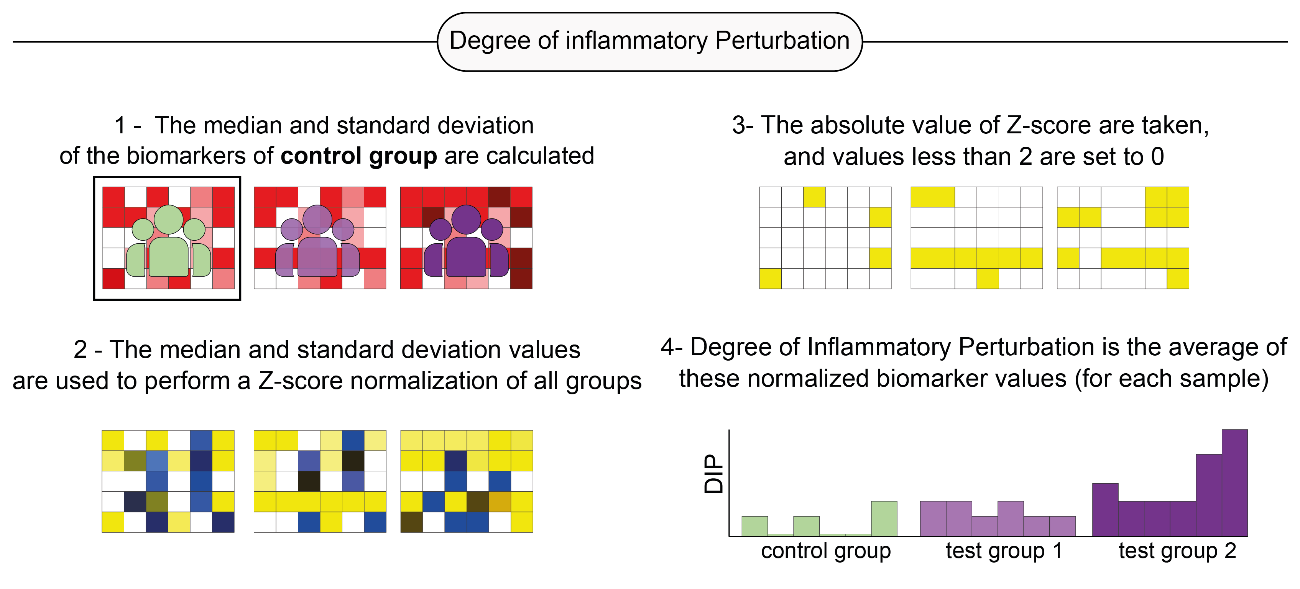
**

**Supplementary Figure 1.** Degree of inflammatory perturbation (DIP) is based on Molecular Degree of Perturbation, but instead of using gene expression, we used biochemical and cellular markers. DIP was calculated using the median and standard deviation of the control group as a starting point. Then, the Z-score was calculated for all groups, a cut-off point was established and, finally, an average disturbance calculation was performed for each sample. This figure was adapted https://mdp.sysbio.tools/about.

**
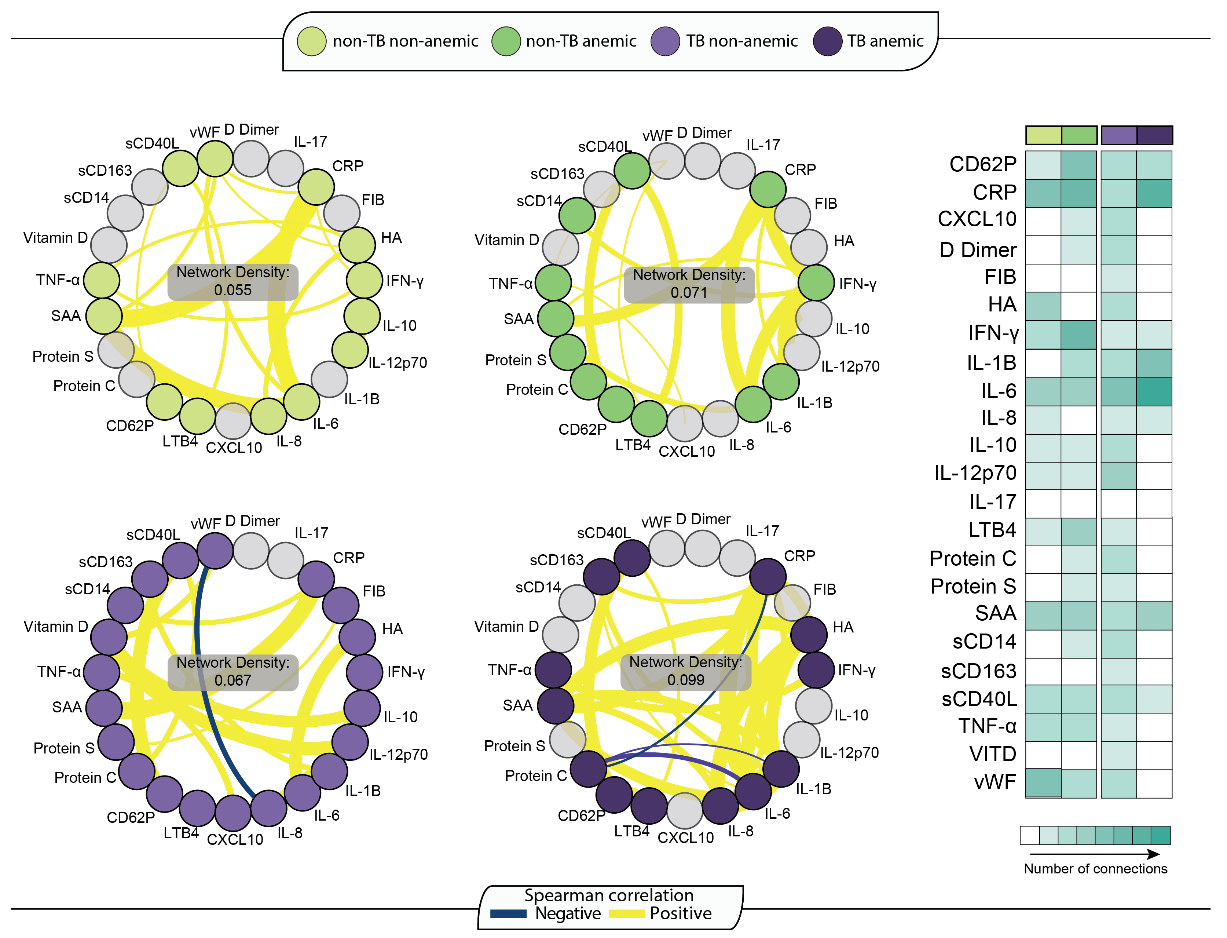
**

**Supplementary Figure 2.** Anemic TB patients have greater interconnectivity between biomarkers. Left: Spearman correlation test between laboratory measurements for each group according anemia and TB status. Blue lines indicate negative correlation, and yellow lines indicate a positive correlation between the linked parameters. All correlations in this chart had p value less than 0.05. Right: Number of connections by biomarker and group.

**Supplementary Table 1. AIDS defining illness**

| AIDS-defining illness | n |
| --- | --- |
| *Candidiasis* of esophagus | 10 |
| *Cryptococcosis* | 5 |
| *Cryptosporidiosis* | 5 |
| *Cytomegalovirus* | 4 |
| *Histoplasmosis* | 3 |
| *Isosporiasis* | 1 |
| *Pneumocystis jirovecii pneumonia* | 22 |
| *Kaposi Sarcoma* | 4 |
| *Toxoplasmosis* | 4 |
| *Mycobacterium tuberculosis* | 58 |
| Wasting syndrome attributed to HIV | 40 |

**Supplementary Table 2. Biomarker levels according to clinical groups.**

|  | **non-TB** | | | **TB** | | |
| --- | --- | --- | --- | --- | --- | --- |
|  | **Non-anemic at baseline (n=34)** | **Anemic at baseline (n=64)** | **P value** | **Non-anemic at baseline (n=12)** | **Anemic at baseline (n=49)** | **P value** |
| D Dimer (mg/L) | 0.74 (0.52-1.31) | 1.17 (0.62-1.77) | 0.062 | 0.45 (0.31-1.11) | 1.54 (1.17-2.20) | **0.003** |
| CRP (mg/L) | 2.77 (1.22-6.62) | 4.44 (1.37-9.88) | 0.256 | 4.07 (1.01-6.99) | 9.30 (2.82-26.2) | **0.043** |
| SAA (mg/L) | 3.85 (1.27-7.29) | 7.28 (2.76-15.6) | 0.09 | 5.00 (1.49-10.1) | 12.5 (2.60-32.7) | 0.218 |
| CD62P (ng/mL) | 56.3 (44.2-69.8) | 51.5 (40.1-71.1) | 0.613 | 40.6 (29.1-43.2) | 55.0 (35.4-80.7) | 0.095 |
| IFN-γ (pg/mL) | 3.47 (2.43-5.60) | 3.93 (1.98-7.31) | 0.852 | 3.15 (1.84-4.06) | 8.62 (3.79-14.5) | **0.009** |
| IL-10 (pg/mL) | 13.9 (9.60-20.3) | 12.3 (8.74-19.4) | 0.351 | 6.91 (6.42-9.87) | 12.4 (8.82-15.9) | **0.037** |
| IL-12p70 (pg/mL) | 1.31 (0.77-2.93) | 0.99 (0.40-2.62) | 0.382 | 0.59 (0.36-0.98) | 1.31 (0.89-2.92) | 0.071 |
| IL-1β (pg/mL) | 0.27 (0.10-0.51) | 0.30 (0.00-0.80) | 0.981 | 0.43 (0.09-0.91) | 0.20 (0.05-0.47) | 0.415 |
| IL-6 (pg/mL) | 2.14 (1.29-2.63) | 2.45 (1.68-3.64) | **0.046** | 1.60 (1.14-1.88) | 2.44 (1.50-4.16) | 0.095 |
| IL-8 (pg/mL) | 11.3 (6.53-20.5) | 11.6 (7.35-17.8) | 0.932 | 5.79 (2.35-8.62) | 9.42 (6.54-14.6) | **0.043** |
| TNF- α (pg/mL) | 16.1 (14.0-20.2) | 20.1 (13.7-27.5) | 0.071 | 12.2 (9.68-14.9) | 22.5 (16.4-29.1) | **0.001** |
| IL-17 (pg/mL) | 0.41 (0.16-0.58) | 0.32 (0.17-0.63) | 0.539 | 0.24 (0.15-0.39) | 0.32 (0.17-0.50) | 0.255 |
| CXCL10 (pg/mL) | 2810 (1588-4240) | 2531 (1821-3837) | 0.963 | 1766 (1148-3014) | 2695 (1867-3972) | 0.109 |
| sCD14 (ug/mL) | 1997 (1634-2306) | 2199 (1796-2461) | 0.247 | 2502 (2088-2770) | 3148 (2665-3755) | **0.015** |
| sCD40L (pg/mL) | 930 (411-1320) | 833 (265-1316) | 0.56 | 272 (93.4-494) | 1027 (514-1536) | **0.001** |
| HA (ng/mL) | 51.3 (37.7-77.7) | 74.5 (45.0-98.9) | 0.179 | 42.8 (15.2-69.9) | 92.1 (49.2-176) | **0.033** |
| sCD163 (ng/mL) | 654 (419-991) | 639 (458-828) | 0.963 | 649 (450-948) | 805 (444-955) | 0.593 |
| LTB4 (pg/mL) | 10.2 (10.2-27.7) | 13.7 (10.2-50.9) | 0.061 | 12.6 (10.2-17.9) | 46.9 (10.2-62.8) | 0.106 |
| FIB (mg/dL) | 761 (505-1403) | 758 (513-1368) | 0.96 | 640 (449-1028) | 1423 (576-2203) | **0.043** |
| vWF (%) | 8510 (6978-11725) | 10059 (8006-12456) | 0.231 | 8935 (8553-11762) | 11120 (9608-13806) | 0.086 |
| Protein S (%) | 3824 (3425-4408) | 3560 (3116-4134) | 0.204 | 3817 (3009-4386) | 4112 (3177-5273) | 0.407 |
| Protein C (%) | 4242 (3351-4770) | 3519 (3220-4125) | 0.054 | 3414 (3071-4072) | 3761 (3163-4385) | 0.522 |
| Vit. D (ng/mL) | 9.35 (6.39-16.6) | 9.65 (4.99-15.0) | 0.913 | 6.32 (4.41-11.9) | 10.4 (5.99-17.0) | 0.134 |

**Table Note:**

Bold font indicates statistical significance, at p<0.05. Data are shown as median and (IQR). Data were compared between groups using the Wilcoxon test for continuous variables. IQR: Interquartile range;
